# Annexin Expression in Cholangiocarcinoma, and Metastatic Pancreatic Ductal Adenocarcinoma “Is it be Helpful for Differential Diagnosis of These Tumors in the Liver?”

**DOI:** 10.30699/IJP.20201.138489.2512

**Published:** 2021-07-06

**Authors:** Bita Geramizadeh, Mahsa Sehat, Azam Mehrmozayan, Ali Reza Shojazadeh

**Affiliations:** 1 *Department of Pathology, School of Medicine, Shiraz University of Medical Sciences, Shiraz, Iran *; 2 *Transplant Research Center, Shiraz University of Medical Sciences, Shiraz, Iran*

**Keywords:** Annexin A1(ANXA1), Annexin A10(ANXA10) and Annexin A13(ANXA13), Cholangiocarcinoma, pancreatic ductal adenocarcinoma, immunohistochemistry

## Abstract

**Background & Objective:**

Differential diagnosis between cholangiocarcinoma (CCA) and metastatic pancreatic ductal adenocarcinoma (PDA) in the liver is difficult and so far, no specific immunohistochemical marker is reported to differentiate these two tumors. Considering the existing literature, the level of expression of Annexins (Annexin A1, 10 and 13) have been studied for differential diagnosis between these two tumors by molecular methods and promising results have been reported. Therefore, in this study, we tried to investigate the immunohistochemical value of these three Annexins for the differential diagnosis of CCA and PDA in the liver.

**Methods:**

The articles that reported the research subject in 10 years (2009-2019), including 45 cases of CCA and 50 cases of metastatic PDA in the liver were evaluated considering the presence or absence of AnnexinA1 (ANXA1), Annexin A10 (ANXA10) and Annexin A13 (ANXA13) expression by immunohistochemistry, were investigated.

**Results & Conclusion:**

This study showed, ANXA1 was positive both in PDA and CCA, ANXA10 was positive in ~60% of PDA cases and ~40% of CCA cases, and ANXA13 was mostly negative in both groups. The best sensitivity was found in cytoplasmic and nuclear ANXA1 (80% and 84%, respectively) to distinguish PDA from CCA and vice versa. The best specificity was observed in ANXA10 and ANXA13 to distinguish PDA from CCA. Also, ANXA13 had the best specificity to distinguish CCA from PDA. Our investigations showed that, ANXA1 probably can classify positive cases correctly, but it cannot discriminate PDA from CCA. ANXA10 had fair sensitivity and specificity to discriminate PDA from CCA. ANXA13 apparently had a high specificity that can help to narrow-down the differential diagnoses.

## Introduction

Malignant liver tumors are either primary or metastatic. Most common primary liver tumors in adults are hepatocellular carcinoma (HCC) and cholangiocarcinoma (CC). Histopathology of CC is very similar to pancreatic ductal adenocarcinoma (PDA) and so far, there has been no immunohistochemical marker to differentiate pancreatic ductal adenocarcinoma and cholangiocarcinoma (1).

Annexins (ANX) are calcium‑dependent phospholipid‑binding proteins comprising 13 members in human (ANXA1‑A11, A13 and A8L1; A12 is unassigned). Annexins are involved in different types of biological processes such as anti‑inflammation, cell differentiation, apoptosis, and proliferation. Aberrant expression of Annexins have been reported in different cancers as tumor promoters and suppressors, but the results in liver cancers are controversial and the level of their expression and usefulness in differential diagnosis in different types of liver malignancies is unclear (2). 

In this study we tried to use Annexins in CC and PDA in the documented cases to find out if these markers can be helpful for differential diagnosis of these two tumors in the liver.

## Patients and Methods

Studying the literature between 2009 and 2019, 95 cases were selected with the definite diagnosis of intrahepatic CCA and metastatic PDAC to the liver in Whipple’s operation specimens from the archives of pathology department of affiliated hospitals of Shiraz University of Medical Sciences, Shiraz, Iran. We selected 45 cases of CC and 50 cases of PDA with definite diagnosis and enough tissue and good quality (no necrosis and proper fixation). Cases with unproven diagnosis or inadequate tissue or extensive necrosis have been excluded from the study. 

The slides were reviewed, and the best representative blocks were selected to be stained with annexins. 

The detailed characteristics of each of the antibodies are shown in Table 1. 

**Fig. 1:**
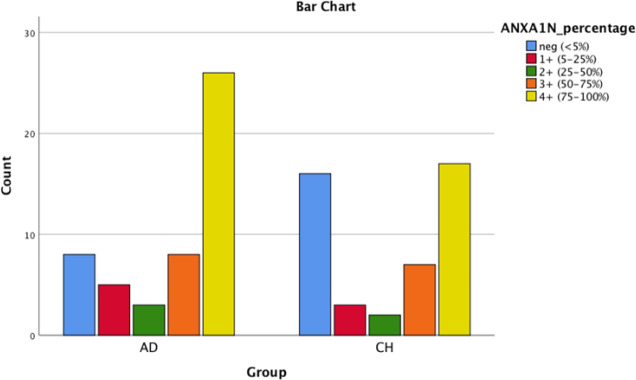
Bar chart shows the comparison in Annexin1 nuclear staining in PDA and CCA

**Fig. 2:**
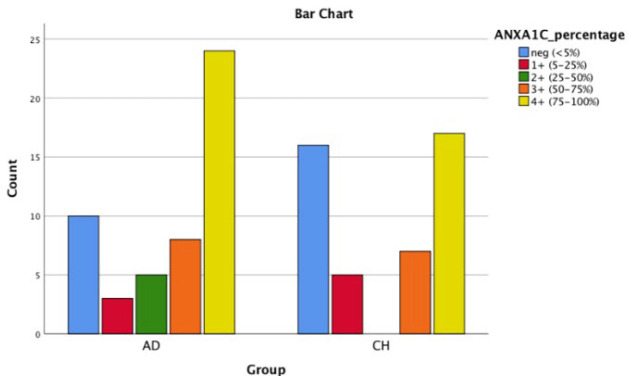
Bar chart shows AnnexinA1 cytoplasmic positivity in PDA and CCA

**Fig. 3:**
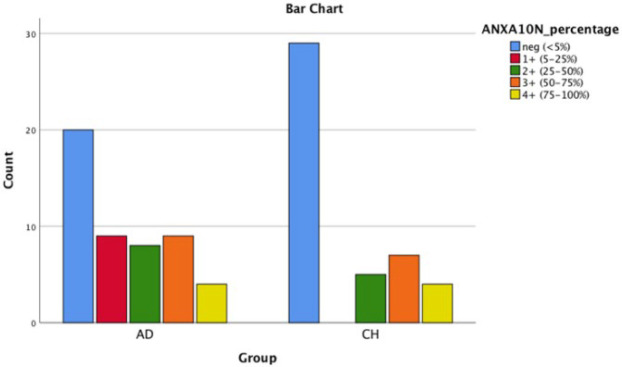
Bar chart shows AnnexinA10 nuclear positivity in PDA and CCA

**Fig. 4:**
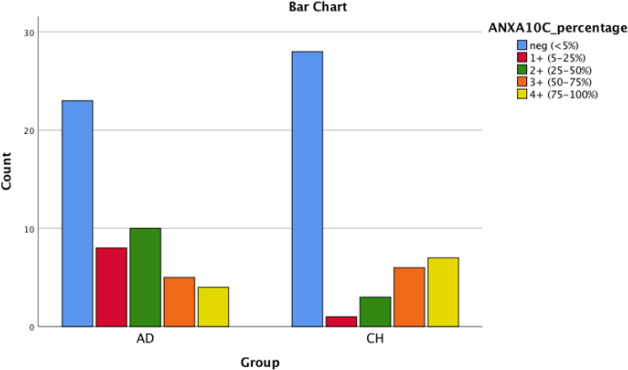
Bar chart shows AnnexinA10 cytoplasmic positivity in PDA and CCA

All the immunohistochemical slides were reviewed by a hepatopathologist and a general pathologist, both of whom were blinded to the final diagnosis (Gold standard). Figures 1, 2, 3 and 4 are sample pictures from our cases. 

Statistical analysis was carried out using statistical package for social sciences (SPSS) Version 22.0. (IBM Corp., Armonk, NY, USA). Qualitative and quantitative variables were described using frequency (percent) and mean ± Standard Deviation (SD), and visualized by box plot. Different variables were compared among the two groups using the Chi-square test for qualitative variables and the Independent t-test for quantitative variables. Also, contingency table was used for obtaining diagnostic test statistics. P-value ≤ 0.05 was considered statistically significant.

**Table 1:** Characteristics of Annexin Antibodies which have been used to differentiate CC and PDA in the liver (ANXA1, ANXA10, and ANXA13)

Antibody	Antigen Retrieval	Lot Number	Description	Brand	Positive control
ANX A1	TE Buffer	04250009	Polyclonal/Rabbit	Invitrogen	Bone marrow/hairy cell leukemia
ANX A10	TE Buffer	4H2835076A	Polyclonal/Rabbit	Invitrogen	Gastric mucosa
ANX A13	TE Buffer	RO8000	Polyclonal/Rabbit	Invitrogen	Duodenal mucosa

## Results & Discussion

A group of 45 patient with CCA and a group of 50-patient with PDAC were qualified for analysis (according to inclusion criteria). Sex distribution was not statistically different (*P*=0.12) but age was significantly higher in PDAC group (*P*=0.008). Demographic and tumoral variables of two groups are depicted and compared in Table 2.

**Fig. 5:**
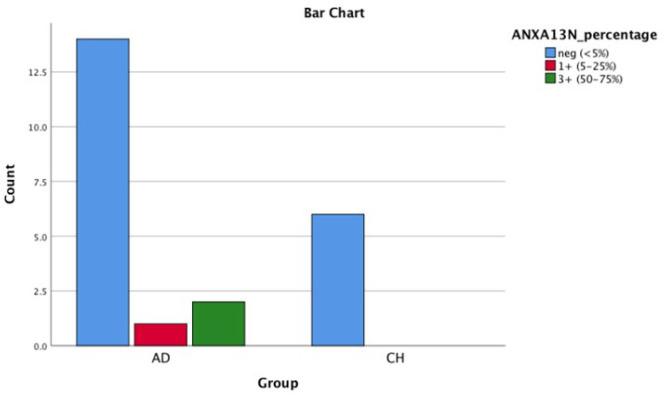
Bar chart shows AnnexinA13 nuclear positivity in PDA and CCA

**Fig. 6:**
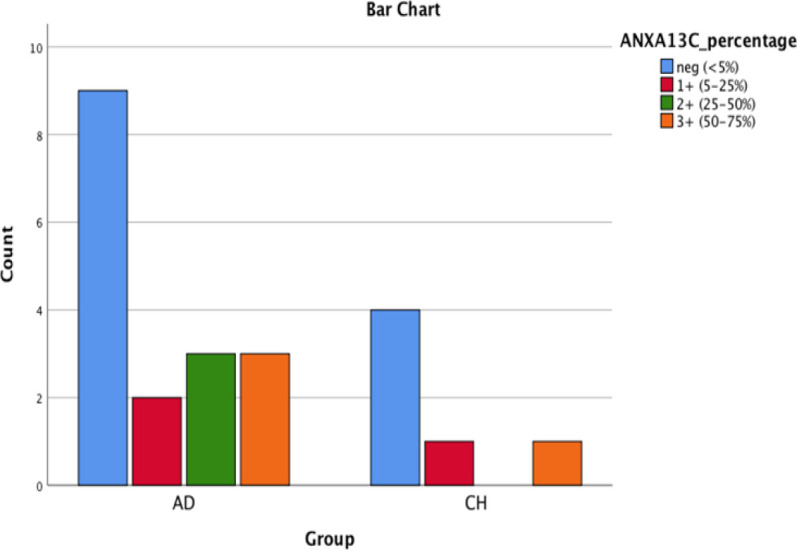
Bar chart shows AnnexinA13 cytoplasmic positivity in PDA and CCA

As shown in Table 3, ANXA10 was significantly more intensely positive in CCA group comparing with PDAC group (*P* (cytoplasmic)=0.023; *P* (nuclear)-=0.035). It should be mentioned that ANXA1 tends to be positive in both groups, ANXA10 stained in ~60% of PDAC cases and ~40% of CCA cases, and ANXA13 was mostly negative in both groups. Figures 1, 2, 3, 4, 5 and 6 depict bar chart comparison of IHC staining percentage result of annexins in PDAC and CCA.

The best sensitivity was related to cytoplasmic and nuclear ANXA1 (80% and 84%, respectively) to distinguish PDAC from CCA and vice versa (Tables 4 and 5).

The best specificity was observed in ANXA10 and ANX13 to distinguish PDAC from CCA (Table 4). Also, ANXA13 had the best specificity to distinguish CCA from PDAC (Table 5). 

**Table 3:** IHC results and comparison in the cases of CCA and PDA (N=Nuclear, C=Cytoplasmic)

	PDAC (n = 50)^1^					CCA (n = 45)^1^					P-value^2^
	neg.	1+	2+	3+	4+	neg.	1+	2+	3+	4+	
ANXA1(N)	16%	10%	6%	16%	52%	35.6%	6.7%	4.4%	15.6%	37.8%	0.28
ANXA1(C)	20%	6%	10%	16%	48%	35.6%	11.1%	0	15.6%	37.8%	0.095
ANXA10 (N)	40%	18%	16%	18%	8%	64.4%	0	11.1%	15.6%	8.9%	0.023
ANXA10(C)	46%	16%	20%	10%	8%	62.2%	2.2%	6.7%	13.3%	15.6%	0.035
ANXA13(N)	82.4%	5.9%	0	11.8%	0	100%	0	0	0	0	0.544
ANXA13 (C)	52.9%	11.8%	17.6%	17.6%	0	66.7%	16.7%	0	16.7%	0	0.731

Abbreviations: (1) PDAC: Pancreatic ductal adenocarcinoma, (2) CCA: Cholangiocarcinoma, (3) IHC: immunohistochemistry, (4) neg. [<5%]; 1+ [5-25%]; 2+ [25-50%]; 3+ [50-75%]; 4+ [75-100%]. ^1^ due to resource limitation, sample size for cytoplasmic and nuclear ANXA13 testing was 17 and 6 for PDAC and CCA, respectively, ^2 ^Chi-square test.

**Table 4:** Diagnostic test results (sensitivity, specificity, PPV and NPP) of ANXAs to distinguish PDAC from CCA (N, Nuclear & C, Cytoplasmic)

	Sensitivity	Specificity	PPV	NPV
ANXA1(N)	84%	35.6%	59.2%	66.7%
ANXA1(C)	80%	35.6%	58%	61.5%
ANXA10(N)	60%	64.4%	65.2%	59.2%
ANXA10 (C)	54%	62.2%	61.4%	54.9%
ANXA13(N)	17.6%	100%	100%	30%
ANXA13(C)	47.1%	66.7%	80%	30.8%

Abbreviations: (1) PPV: positive predictive value, (2) NPV: negative predictive value, (3) PDAC: Pancreatic ductal adenocarcinoma, (4) CCA: Cholangiocarcinoma.

**Table 5:** Diagnostic test results (sensitivity, specificity, PPV and NPP) of ANXAs to distinguish CCA from PDAC

	Sensitivity	Specificity	PPV	NPV
Nuclear ANXA1	64.4%	16%	40.8%	33.3%
Cytoplasmic ANXA1	64.4%	20%	42%	38.5%
Nuclear ANXA10	35.6%	40%	34.8%	40.8%
Cytoplasmic ANXA10	37.8%	46%	38.6%	45.1%
Nuclear ANXA13	0%	82.4%	0%	70%
Cytoplasmic ANXA13	33.3%	52.9%	20%	69.2%

Abbreviations: (1) PPV: positive predictive value, (2) NPV: negative predictive value, (3) PDAC: Pancreatic ductal adenocarcinoma, (4) CCA: Cholangiocarcinoma.

One of the common challenges in the pathology of liver tumors is the differential diagnosis between CCA and metastatic PDA. So far there has not been any useful immunohistochemical marker to differentiate these two tumors especially in small liver biopsies. There are very few studies in the literature with proven useful biomarkers for this differential diagnosis. We considered Padden* et al.* study regarding the usefulness of Annexin A1, 10 and 13 by proteomics to differentiate CCA in the liver from metastatic PDA to the liver (3). So, we tried to use the same markers by immunohistochemistry on the tissues of the liver tumors to find out their role in differential diagnosis of hepatic CC and liver metastasis of PDA. 

**-Annexin A1:** In the previous report, it has been shown that ANXA1 is linked to tumor development and progression (4, 5, 6). It has been shown that that ANXA1 downregulated in esophageal, gastric, and CC and upregulated in PDA (7). 

Wang* et al.* (2010) studied the expression of ANXA1 amongst 61 cases of hilar cholangio-carcinoma. They showed that ANXA1 was not expressed in 55.7% of cases and its downregulation was linked to lymph node metastases, histopathologic grade, recurrence, and poor prognosis (8). In a study on 68, in 2013 Hongsrichan* et al.* (2) showed that ANXA1 was expressed in more than 90% of patients with CC. 

Our results for CC was different with Wang* et al.* (2010) (8) study and similar to Hongsrichan* et al.* (2013) (2) study. We showed that more than 60% of cases with confirmed diagnosis of CC were positive for ANXA1. However, in Hongsrichan’s study all samples were associated to an *Opisthorchis viverrini* infection, which might affect the protein expression profile of the cancerous cells. None of our cases showed this association (2).

Liu* et al.* (2016) studied ANXA1 expression in 162 cases of PDA. They showed that decreased expression of ANXA1 is associated with poor differentiation, lymph node metastasis, advanced TNM stage and poor survival of PDAC. These findings were again supported by ANXA1 gene knockdown assessment, which inhibited cell proliferation through G1 phase cell cycle arrest and by modulating MMP-9 activity and its inhibitor TIMP-1 significantly increased PDAC cell migration and invasion (6). In contrast, Gao* et al.* (2014) showed that ANXA1 was overexpressed in PDA. However, their sample size was very small (4 patients) (7). In another study by Bai* et al.* (2004) reported that ANXA1 overexpressed in PDA compare to its normal adjacent tissue (9). Padden* et al.* compared 73 patients with cholangiocarcinoma and 96 patients with PDAC (primary: n=78, metastatic: n=18) regarding ANXA1, 10 and 13 expression (3). Their results were similar to the above-mentioned studies (7-9), i.e. ANXA1 was overexpressed in PDA. They concluded that ANXA1 is a reliable biomarker for differential diagnosis between PDA from CC by a sensitivity of 84% and a specificity of 85% (3). 

In our study regarding PDA, we showed that ~80% of the cases with confirmed diagnosis of PDA express ANXA1, which was similar to the results of Gao* et al.* (2014) (7), Bai* et al.* (2004) (9) and Padden* et al.* (2015) (3). Similar to Padden’s study, we showed that ANXA1 had the best sensitivity to discriminate PDA from CCA and vice versa; but, in our study, the specificity was low. This means that ANXA1 probably can classify positive cases correctly, but it cannot discriminate PDA from CCA.

**ANXA10:** This marker is reported to be overexpressed in PDA. In some reports, ANXA10 had a sensitivity of 90% and specificity of 66% in discriminating PDA from cholangiocarcinoma (3). Similarly, in the study by Liu* et al.* (2013), 78% of primary PDA tumors, 83% of metastatic PDA tumors and only 17% of cholangiocarcinomas showed positive ANXA10 staining. ANXA10 has reported as marker with an excellent specificity but relatively low sensitivity for tumors with pancreatobiliary origin (6). 

Kälsch* et al.* (2017), tested various markers for the discrimination between intrahepatic cholangiocarci-noma and metastases of PDA, comprising Annexin A1, Annexin A10, MUC5 AC, CK17, and N-Cadherin. They showed that ANXA10 was positive in 25% of CCA cases and 85% of the cases with the confirmed diagnosis of PDA. The authors recommended ANX-A10 as the best immunohistochemical biomarker to discriminate between CCA and PDA (1).

Our results were different and expressed that ANXA10 was positive in ~60% of the cases with the diagnosis of PDA and ~40% of the cases of CCA. The sensitivity and specificity of ANNA10 to discriminate PDA from CCA was ~60% and ~65%, respectively. So, our study did not confirm ANXA10 as the best biomarker to distinguish PDA vs. CCA.

**ANXA13: **There are quite a few studies about ANXA1 and ANXA10, but to the best of our knowledge there is only one study in field of PDA and ANXA13. In the study by Padden* et al.* higher expression of ANXA13 was found in CCA being compared with PDA with a sensitivity of 84% and a specificity of 55%. ANXA13 has been positive in 8 out of 18 PDA cases; therefore, ANXA13 was not a good biomarker to distinguish CCA from PDAC. (10)

In our study, the majority of the cases in both groups were negative for ANNXA13. However, it didn’t have a good sensitivity to distinguish CCA from PDA and vice versa, but its high specificity can be helpful to narrow-down the differential diagnoses. 

## Conclusion

In summary, ANXA1 had the best sensitivity to detect PDA and CCA, but the specificity was low. Therefore, ANXA1 probably can classify positive cases correctly, but it cannot discriminate PDA from CCA to narrow-down the list of differential diagnoses. ANXA10 had fair sensitivity and specificity to discriminate PDA from CCA. So, we cannot represent ANXA10 as the best biomarker to distinguish PDA vs. CCA. ANXA13 apparently had a high specificity that can help to narrow-down the differential diagnoses.

The results of our study showed that ANXA1, A10 and A13 can be considered as good immunohis-tochemical biomarker candidates to differentiate CCA and PDA, however they should be interpreted in a panel in conjunction with the clinical and imaging findings.
